# Nutritional Status and Obesity Prevalence in People with Gender Dysphoria

**DOI:** 10.3934/publichealth.2014.3.137

**Published:** 2014-08-06

**Authors:** María Victorina Aguilar Vilas, Gabriela Rubalcava, Antonio Becerra, María Carmen Martínez Para

**Affiliations:** 1 Biomedical Science Department, Alcala University, Alcalá de Henares, Madrid 28806, Spain.; 2 Nutrition Department, Guadalajara Campus, Valle de Atemajac University, Guadalajara 28014, Mexico.; 3 Endocrinology and Nutrition, Gender Unit, Ramón y Cajal Hospital, Madrid 28806, Spain.

**Keywords:** nutritional status, lifestyle, eating behavior, obesity, transgender

## Abstract

Gender dysphoria is a condition that involves a failure to adapt and a body dissatisfaction that makes these individuals especially susceptible to eating disorders. The aim of this paper was to ascertain the nutritional status, dietary behaviour and lifestyle and their effect on overweight/obesity prevalence of the people with gender dysphoria.

**Methods:**

A longitudinal study on 157 individuals from the Gender Disorder Unit at the Ramón y Cajal Hospital (Madrid) who are undergoing hormonal treatment has been carried out. Usual dietary intake, physical activity habits and socioeconomic parameters were evaluated. The anthropometric parameters determined were weight, height, body-mass index (BMI), waist and hip circumference and body fat content.

**Results:**

The mean of the population eats a large number of servings of food, which leads to high levels of energy intake: 3,614.32 ± 1,314 kcal/day. These intakes are related to the physical activity performed. The average diet among this population is unbalanced, with a high consumption of fats, especially saturated fats and cholesterol. The breakfast is skipped by 16% of the population. Together with cross-hormone treatment, this dietary habitsand lifestylelead to an increase in body fat, especially in the female to male group whose overweight andobesity prevalence increase (22.72% *vs* 34.85%).

**Conclusion:**

This population suffers a change of their nutritional status due to a variation in their eating behaviour and lifestyle. This increase in the obesity prevalencemake it susceptible to chronic diseases and cardiovascular disorders. It is therefore necessary to include nutrition education courses in the comprehensive treatment programme (anatomical, psychological, etc.) for these individuals.

## 1. Introduction

Gender Identity Disorder (GID) or gender dysphoria is a condition characterized by a strong and persistent identification with the opposite sex, a persistent discomfort with one's own gender and a sense of inappropriateness in one's gender role, leading to deep psychological distress and significant changes in the social or occupational sphere or in any other important aspect of functioning [1]. The prevalence of GID in Spain is estimated at a total of 2,087 transsexuals (1,480 male to female and 607 female to male), with an estimated annual incidence of 61 people who could request assistance and follow the diagnostic and therapeutic process [2].

These social and psychological changes, coupled with concern that some of these individuals have regarding their body image, makes them particularly susceptible to eating disorders [3]. Psychosocial changes have an impact on the food consumption pattern. These people has inadequate nutrients intake, they may eat more or skip meals leading to malnutrition, obesity and other nutritional complications.

The aim of this study is assess the nutritional status and to track the overweight/obesity prevalence and to study the dietary behavior and lifestyle of the people with gender dysphoria to explain the body changes. This will allow us to detect alterations in nutritional status associated with chronicle disease risk, and so be able to elaborate a protocol of nutritional advice that serves to promote the health of these individuals.

## 2. Materials and methods

### 2.1. Study population

The sample for our analyses included 157 transgender healthy subjects with no metabolic diseases, who were divided into two groups: FTM (female to male) (45%) and MTF (male to female) (55%). This population has a mean age of 32.9 ± 9 years old. The age of the FTM group was lower than that of the MTF group (30.4 *vs* 34.6 years, respectively; *P* = 0.004). The nationality of these subjects was: 73.9% Spanish, 22.3% South American, 2.5% Asiatic and 1.3% African. For most age-related comparisons, participants were randomized into four groups according to age (< 19, 20–39, 40–49 and > 50 years). The population was recruited among the patients of Gender Unit at the Ramón y Cajal Hospital, which is the Reference Unit in the Autonomous Region of Madrid. Each participant, who received psychiatric consultation before and during their hormonal treatment, agreed to take part in our investigation by signing an informed consent before entering the study, and the ethical standards of the Hospital were met. Each individual was examined by medical personnel and surveyed with a comprehensive questionnaire that they completed with trained researchers (including information about smoking habits, medical history and current use of medications, lifestyle, eating habits, and socioeconomic status). The inclusion criteria in this study were: patients of both genders, any nationality and aged over 18 years old, patients who wished to carry out sex reassignment treatment, were in the process of doing so or who had completed it, patients referred by the doctor heading the Gender Disorder Unit and the patient's consent for participation in the study.

### 2.2. Hormonal treatment

Conjugated oestrogens (EEC) orally (4 to 6 tablets/day = 2.4 to 3.6 mg/day) or estradiol valerate 2 mg/day orally and cyproterone acetate orally, 50 mg/12 hours, were used in the feminizing treatment for MTF transsexuals. For FTM transsexuals, testosterone was used in different forms: as a gel (50 mg/day) or testosterone undecanoate, 1,000 mg/3 months *i.m.* The period of hormonal treatment was 20 ± 30.88 months.

### 2.3. Measurement of diet

Usual dietary intake was assessed with the use of a 169-item quantitative food-frequency questionnaire and a 24-h dietary recall to obtain detailed information about foods, preparation methods, and the ingredients used in preparation. To estimate the portion sizes of every food item consumed, the subject referred to a photo-book [4]. All questionnaires were administered by trained dieticians. Each food and beverage was then coded and analyzed for its content of energy and other nutrients using DIAL software. An appropriate intake of nutrients was determined within the recommended nutrient intakes to Spanish population. No subject had lactose intolerance during his/her lifetime.

### 2.4. Physical activity assessment

For physical activity assessment, subjects completed a physical activity-recall questionnaire. They were asked to record whether they were sleeping, sitting, standing, or watching television during each hour of the day, during the previous week. Subjects noted the time of day they started each new activity, and the effort (light, moderate, or vigorous). The total hours spent on each activity and the physical activity level (PAL) was computed as the total energy expended over 24 h divided by 24. To estimate the basal metabolic rate (BMR) we used predictive Harris-Benedict formula. Multiplying the PAL by the BMR produced the total energy expenditure. The same researcher instructed the subjects on the use of these physical activity records and inspected the completed forms.

### 2.5. Anthropometric determination

Measurements of height using a stadiometer and weight on a digital scale while wearing light clothing without shoes were used to calculate body-mass index (BMI). The waist and hip circumferences were measured at the level of the umbilicus and the widest area around the buttocks, respectively. According to World Health Organization criteria [5], the BMI is categorized into three groups as normal (< 25 kg/m^2^), overweight (25–< 30 kg/m^2^), and obese (≥ 30 kg/m^2^). After taking into account the waist circumference was considered, subjects with waist circumferences of at least 80 cm and smaller than 88 cm were classified as overweight and those with a waist circumference of at least 88 cm were classified as obese [5]. Fat mass was determined by bioelectrical impedance (OMRON BF 300 monitor). All these measurements were undertaken by the same individual to avoid data variability from measurement techniques. Anthropometric data were noted at baseline and after a period of treatment.

### 2.6. Statistical analysis

Analysis was performed with SPSS 17.0 (SPSS Inc., Chicago, IL, USA). Subgroups were analyzed by gender and age and statistical results are presented as means ± SD. The Student's *t*-test and one-way ANOVA were used for differences between groups. The Pearson linear correlation analysis was used to explore the association between nutrient intakes and the factors analyzed.

## 3. Results

### 3.1. Anthropometric characteristics of samples studies

The anthropometric parameters analyzed are shown in Table 1. The transgenders included in this study present a mean baseline BMI of 24.0 ± 5.0 kg/m^2^. No significant differences were observed when the two groups were considered separately, although MTF group had a lower BMI than FTM group (*P* = 0.71). 4.39% of MTF and 6.06% of FTM group were underweight, as they have a BMI < 19 kg/m^2^, and 12% of MTF *vs* 15.15% of FTM were overweight (BMI > 25 kg/m^2^). 5.49% of MTF and 7.57% of FTM was obese (BMI > 30 kg/m^2^) [5]. After hormonal treatment, the mean BMI was 24.1 ± 4.1 kg/m^2^ (*P* = 0.12 *vs* baseline), with more extensive differences between the two groups at baseline: the FTM group presented higher values (25.1 ± 4.6 kg/m^2^, *P* = 0.06). The eating practices and hormonal treatment affect significantly on BMI (*r* = 0.414 and *P* = 0.029). 2.02% and 3.03% of individuals of MTF and FTM, respectively, were underweight; 10.99% of the individuals MTF were overweight and 5.49% presented obesity after the treatment. In the FTM group the data were: 24.24% of individuals suffered overweight and 10.61% obesity. In both groups, there was a tendency towards larger stores of body fat (Table 1).

**Table 1.** Anthropometric characteristics of the patients.

Variable	Baseline data				Post-treatment data				^b^*P*
	All (*N* = 157)	MTF (*N* = 91)	FTM (*N* = 66)	^a^*P*	All (*N* = 157)	MTF (*N* = 91)	FTM (*N* = 66)	^a^*P*	
Weight (kg)	67.8 ± 13.4	69.5 ± 14.2	65.8 ± 12.1	0.14	68.1 ± 12.3	68.8 ± 11.9	66.9 ± 12.9	0.45	0.82
Height (m)	1.7 ± 0.1	1.7 ± 0.06	1.6 ± 0.07	< 0.001	1.7 ± 0.1	1.7 ± 0.06	1.6 ± 0.07	< 0.001	
BMI (kg/m^2^)	24.0 ± 5.0	23.8 ± 4.3	24.2 ± 5.8	0.71	24.1 ± 4.1	23.5 ± 3.7	25.1 ± 4.6	0.06	0.12
Waistcircumference (cm)	80.7 ± 10.8	79.9 ± 10.2	81.6 ± 11.5	0.43	80.0 ± 10.1	79.3 ± 9.1	81.3 ± 11.6	0.31	0.99
Hip circumference (cm)	98.8 ± 10.5	97.4 ± 10.0	100.5 ± 10.8	0.13	98.1 ± 9.0	97.1 ± 9.0	100.0 ± 8.8	0.11	0.47
Body fat (%)	27.9 ± 10.7	28.5 ± 11.7	25.5 ± 5.8	0.44	28.9 ± 10.2	29.7 ± 11.7	26.8 ± 4.5	0.34	0.20

^a^*P*: Significance between group transgender, ^b^*P*: Significance between baseline and postreatment data.

### 3.2. Physical activity

**Figure 1.**
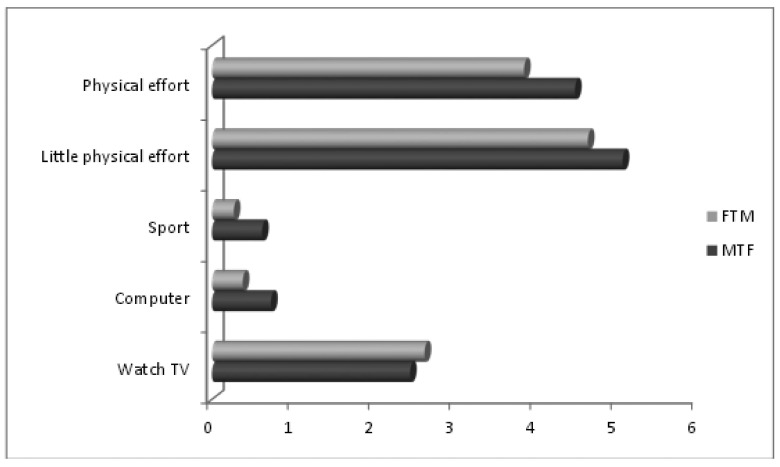
Hours dedicated to various leisure activities every day.

Although 56% of patients wanted to lose weight at some point and 42% felt that they were overweight, this type of population does not usually engage in daily physical activity. They devote more time (4 to 5 hours) to activities that require little physical effort (Figure 1). This activity is influenced by the type of work they do. Civil servants therefore spend the most time on low-effort activities while those who do not work spend more time watching TV or using a computer. Among the various employment groups considered, it is senior executives who outside working hours spent more time playing sports, perhaps to compensate for the inactivity in their profession and to improve body image. No significant correlation coefficients were found between PAL and BMI (*r* = 0.1705). Both groups can be considered as sedentary because the PAL was < 1.70 (range: 1.21–2.08) [6].

### 3.3. Nutrient intakes during hormonal treatment

Although there are results of basal food intakes, this item only includes data during hormonal treatment justifying the body changes of the studied population. Regardless of the two groups in which it was classified, the transgender population studied consumes a mean of 3,614.3 ± 1,314 kcal (range: 1,660–7,752 kcal) with significant deference's (*P* = 0.14), FTM group consumed a diet 11.85% higher in energy (Table 2). If the groups are stratified according to the age ranges in the tables of recommended intake for the Spanish population [7]. FTM group presented higher caloric intakes than MTF group in all the subgroups studied, and these differences are significant in the 18–19 and 50–59 years old age groups (*P* < 0.05). Female transgenders in the two groups mentioned above have a diet more suited to their energy needs, with average intakes of 2,681 and 2,023 kcal.

Caloric intake is related to physical activity. MTF group engaging in increased physical activity (*r* = 0.43, *P* = 0.016), either because their work is intense, or because they play sports, are therefore those with the highest calorie intake. The opposite is true of the FTM group, as the highest correlations are between energy intake and hours spent watching television (*r* = 0.41, *P* = 0.006).The diet consumed by the transgender population is unbalanced. In both cases the diets are hyperlipidic (38.60% MTF *vs* 39.90% FTM), hyperproteic (16.45% MTF *vs* 17.58% FTM) and therefore hypoglucidic (44.17% MTF *vs* 41.59% FTM).

There was not relationship between consumption of macronutrients and age and treatment period. Although the participants propensity for energy-dense and micronutrients poor foods was high, the large size of ingested rations entails a high consumption of nutrients. The levels of fibre consumed are therefore 35.6 g/day in the MTF group and 35.5 g/day in the FTM group (Table 2). These intakes are higher than those specified in Dietary Goals of the Spanish Community Nutrition Society (SENC) [8] or the WHO [9]. The same is true of cholesterol. The intake in both groups exceeds the recommended daily intake (DRI) (< 300 mg/day), especially in the FTM group, which is 34% higher than in the MTF group. Only the MTF group over 50 years old complies with the recommended intake (Table 2).

**Table 2.** Nutrient intake and physical activity (expressed as PAL) in the transsexual population studied.

Nutrients	MTF (*N* = 91)					FTM (*N* = 66)					*P* value
	Mean	< 19(*N* = 14)	20–39(*N* = 38)	40–49(*N* = 24)	> 50(*N* = 15)	Mean	< 19(*N* = 8)	20–39(*N* = 29)	40–49(*N* = 19)	50(*N* = 10)	
PAL	1.41 ± 0.46	1.96 ± 0.52	1.81 ± 0.46	1.34 ± 0.23	2.08 ± 0.43	1.38 ± 0.43	1.28 ± 0.20	1.21 ± 0.19	1.41 ± 0.43	1.58 ± 0.42	0.25
Energy(kcal)	3354.63±1439.9	2681 ± 794.8	3547 ± 1507.7	3265 ± 1320.8	2023 ± 420.1	3805.6 ± 1530.7	4111 ± 977	3606 ± 1660	4116 ± 1452.08	3900 ± 990.6	0.14
Proteins(g)	1380.5 ± 79.0	101 ± 61.6	145 ± 86.72	138 ± 58.8	195 ± 33.2	163.9 ± 67.6	156 ± 53	156 ± 72.15	176 ± 61.1	214 ± 82.02	0.91
Lipids(g)	147.36 ± 82.0	106 ± 60.4	162 ± 88.9	123 ± 50.7	83 ± 14.03	175.0 ± 92.2	193 ± 39	165 ± 100	194 ± 90.7	143 ± 24.7	0.12
Cholesterol(mg)	494 ± 261.9	322 ± 270.1	519 ± 278.4	519 ± 205.2	289 ± 99.9	664.6 ± 425.9	598 ± 242	678 ± 518.2	667 ± 266.3	562 ± 69.29	0.24
SFA (g)	44.3 ± 24.1	33 ± 27.4	48 ± 24.4	39 ± 23.8	23 ± 3.0	55.1 ± 29.6	61 ± 18	51 ± 31.9	63 ± 28.1	41 ± 4.59	0.57
MFA (g)	67.9 ± 47.37	52 ± 23.4	75 ± 53.2	54 ± 19.51	38 ± 7.96	77.2 ± 45.8	92 ± 12	72 ± 52.4	85 ± 39	64 ± 10.5	0.33
PFA (g)	23.2 ± 12.4	14 ± 5.37	25 ± 13.08	20 ± 9.87	16 ± 7.56	28.9 ± 16.9	26 ± 9	28 ± 14.9	32 ± 22.6	24 ± 8.62	0.7
Carbohydrates(g)	345.6 ± 162.4	320 ± 4.94	354 ± 170.4	378 ± 160.1	200 ± 65.9	368.3 ± 139.8	407 ± 123	352 ± 142	384 ± 147.4	422 ± 103.2	0.46
Fibre(g)	35.6 ± 26.5	19 ± 10.9	37 ± 28.4	39 ± 25.9	24 ± 7.91	35.5 ± 15.8	40 ± 19	33 ± 12.9	41 ± 20.1	30 ± .35	0.84

**Table 3.** Micronutrient intake of the population studied.

Micronutrients	MTF					FTM					*P* value	DRIMen	DRIWomen
	Mean	< 19	20–39	40–49	> 50	Mean	< 19	20–39	40–49	> 50			
Minerals													
Calcium (mg)	1441.0 ± 1170.5	835 ± 572	1481 ± 1302	1654 ± 851	935 ± 193	1579.1 ± 889.4	1834 ± 810	1346 ± 660	1680 ± 505	3988 ± 2971	0.51	800–1000	800–1000
Iron (mg)	24.2 ± 3.4	19 ± 7	26 ± 2.6	21 ± 10	15.5 ± 2.3	24.9 ± 11.5	27 ± 9	24 ± 13	26 ± 8	20 ± 0.21	0.86	10–15	10–18
Iodine (mg)	176.0 ± 128.1	91 ± 48	176 ± 134	225 ± 128	115 ± 19	196. ± 112.0	168 ± 67	172 ± 83	218 ± 76	475 ± 40	0.40	125–145	110–115
Magnesium (mg)	495.1 ± 272.1	299 ± 145	502 ± 285	564 ± 242	395 ± 204	541.1 ± 185.5	602 ± 206	492 ± 165	590 ± 183	820 ± 224	0.32	350–400	300–350
Zinc (mg)	17.0 ± 11.7	13.8 ± 12.1	18 ± 13	16 ± 5	12 ± 5	19. ± 9.3	20 ± 7	19 ± 10	21 ± 7	25 ± 6	0.17	15	15
Selenium (mg)	157.5 ± 84.9	91 ± 26	160 ± 87	172 ± 97	137 ± 49	170.6 ± 73.8	137 ± 56	166 ± 80	185 ± 66	190 ± 66	0.42	55	55
Sodium (mg)	4368.1 ± 1863.8	5130 ± 2447	4522 ± 1992	4047 ± 1435	3023 ± 439	5021.4 ± 2140.8	5303 ± 1753	4733 ± 2206	5409 ± 2224	5973 ± 1165	0.12	< 2400	< 2400
Potassium (mg)	5370.4 ± 3316.4	3143 ± 2085	5508 ± 3493	6343 ± 3082	3166 ± 1141	6003.2 ± 2238.8	6528 ± 2149	5461 ± 1810	6573 ± 2523	9072 ± 3038	0.27	2000	2000
Phosphorus (mg)	2418.7 ± 1369.7	1588 ± 702	2502 ± 1467	2629 ± 1219	1624 ± 684	2763.2 ± 1023.1	2619 ± 820	2560 ± 1904	2996 ± 829	4445 ± 2271	0.16	700	700
Vitamins													
Vitamin B1 (mg)	2.4 ± 1.3	2 ± 1.3	3 ± 1.1	3 ± 0.97	3 ± 0.57	2.7 ± 1.2	3 ± 1	3 ± 1.26	3 ± 1.22	2 ± 0.63	0.39	1.1–1.2	0.8–0.9
Vitamin B2 (mg)	3.2 ± 1.9	2 ± 0.9	3 ± 0.3	4 ± 1.6	4 ± 0.8	3.4 ± 1.5	4 ± 1	3 ± 1.7	3 ± 1.0	4 ± 0.3	0.49	1.4–1.8	1.1–1.5
Niacin (mg)	61.8 ± 31.7	45 ± 18.8	65 ± 6.5	61 ± 28.1	44 ± 22.4	71.6 ± 33.6	68 ± 26	70 ± 37.9	65 ± 29.1	61 ± 5.7	0.15	16–20	12–17
Vitamin B6 (mg)	3.8 ± 2.0	3 ± 1.1	4 ± 1.0	4 ± 0.4	2 ± 1.8	4.1 ± 1.7	4 ± 2	4 ± 1.8	4 ± 1.6	4 ± 0.5	0.43	1.6–2.1	1.6–2.1
Vitamin B_12_ (µg)	15 ± 10	6 ± 0.0	14.1 ± 1.0	20.3 ± 7.7	7 ± 0.0	24 ± 3.31	10.3 ± 6.0	27 ± 4.0	23.8 ± 2.4	20.8 ± 5.6	0.11	2	2
Biotin (µg)	48.2 ± 25.9	25 ± 1.6	49 ± 4.0	60 ± 20.8	34 ± 15.7	52.6 ± 29.6	48 ± 16.0	51 ± 34.7	12 ± 3.9	17 ± 2.1	0.45	30	30
Folic Acid (µg)	513.8 ± 369.3	302 ± 110.3	536± 5.36	558 ± 306	325 ± 136.6	547.5 ± 256.3	646 ± 336.0	502 ± 235.6	612 ± 288.1	570 ± 39.5	0.60	300–400	300–400
Vitamin C (mg)	292.1 ± 205.2	148 ± 89.5	289 ± 28.9	411 ± 31.7	144 ± 71.2	325.8 ± 264.6	499 ± 49.0	267 ± 168.2	384 ± 33.8	454 ± 28.5	0.49	60	60
Retinol (µg)	2650.9 ± 2710.8	276 ± 16.7	1694 ± 169.4	2058 ± 194.8	177 ± 36.3	2995.7 ± 5671.0	525 ± 198	2653 ± 713.7	1785 ± 286.7	698 ±55.0	0.72	1000	800
Vitamin D (µg)	5.8 ± 4.9	4 ± 0.7	6 ± 0.6	5 ± 3.5	2 ± 0.4	5.4 ± 4.9	4 ± 3	6 ± 4.0	4 ± 2.3	17 ± 1.9	0.69	5–15	5–15
Vitamin E (mg)	20.0 ± 11.7	11 ± 2.8	21 ± 2.6	19 ± 1.1	14 ± 4.7	22.5 ± 11.1	24 ± 9.0	23 ± 11.7	22 ± 11.4	20 ± 5.7	0.28	10–12	10–12

As regards the type of fat consumed by the two groups, neither the objectives of the WHO nor the intermediate objectives of the SENC are met. The MUFA intake (17.94% MTF *vs* 17.72% FTM) is greater than or equal to the objectives of the SENC. SFA intake (11.59% MTF *vs* 12.54% FTM) almost doubles the levels considered healthy, and MUFA (6.20% MTF *vs* 6.74% FTM) do not cover the SENC nutritional objectives.

The amount of micronutrients consumed is shown in Table 3. This table shows that the average intakes of FTM and MTF groups are higher than the recommended levels for both vitamins and mineral elements; however, when the subgroups are considered according to age there are some shortfalls in intakes of Ca, I, Mg Zn and Vitamin D in the MTF group under 19 years old and of vitamin D in FTM group under than 19 years old and 40–49 years old, as well as intakes of Fe, Zn, K and retinol in MTF group over 50 years old. In other words, the groups with the lowest calorie intakes are most likely to have deficient micronutrient intakes, which could be related to possible chronic diseases over time [10]. Statistically significant correlations were also obtained in FTM group between age and consumption of some minerals: older members have a greater consumption of calcium (*r* = 0.334, *P* = 0.014), iodine (*r* = 0.36; *P* = 0.008) and phosphorus (*r* = 0.29, *P* = 0.041).

The percentage of Daily Recommended Intake (DRI) covering the diet of the study population was not included in any of the tables because its characteristics do not match those of the standard population considered in the consumption tables for the Spanish population as a result of their sex change, and the recommended intakes for both sexes were deemed to have been covered when the above comparison was made.

Intake of these nutrients is distributed among 2 to 3 meals a day (54% of population), with the most common being breakfast, lunch and dinner. However, a large percentage of population studied (16%) usually skip breakfast (Table 4). When asked about the cause of this omission, they answer that this is due to being in a hurry in the morning, because they are reluctant to get up early to prepare and/or eat a healthy breakfast, due to not having to work on preparing breakfast or to prevent weight gain. Interestingly, 1% of population eats food only once a day.

Although 79% of the studied population tends to eat outside the home, only 16.5% assist catering services 1 or 2 times per week. Thus, the 63% eat outside home but does not use catering services.20.5% of transgender eat alone and they are those who eat somewhat less energy diets.

**Table 4.** Meals/day consumed by the study population.

Meals/day	%
Breakfast, Lunch, Dinner	39.0
Breakfast, Lunch, Snack, Dinner	15
Lunch, Dinner	14
Breakfast, Mid Morning, Lunch, Snack, Dinner	13
Lunch, Snack, Dinner	1
Breakfast, Lunch	1
Lunch	1

## 4. Discussion

The eating habits and the crossed hormonal treatment entail a modification in nutritional status. In fact, numerous studies have shown that oestrogen therapy changes body composition, with a loss of lean tissue and a gain in fat mass. The role of the androgens is still controversial, because testosterone induces the appearance of its own receptors in fat tissue, but treatments with dihydrotestosterone, as in our study, have no effect on fat redistribution.

The subjects with GID studied are individuals who, according to the data obtained in the surveys conducted, have problems with their body image during the hormone treatment phase and even beforehand. This makes them introverted, withdrawn and unsociable, as well as feeling rejected by the rest of the population. This alteration of their emotional state causes dietary disorders which can justify the increase in the prevalence of overweight and obesity. These individuals with low self-esteem attempt to use food to relieve the rejection that they inspire or believe they inspire among the rest of the population. Under these circumstances, they consume large amounts of food high in fat, very high energy diets, with a large number of energy-dense foods. The study population associates these foods with pleasant occasions such as family parties [11]. The MTF group is, due to adopting the female role, more concerned with their image and consumes slightly less caloric diets. For this reason, this group presents the lower prevalence of Overweight/obesity. Similar results were obtained by Sivakami and Veena [12]. This intake is higher than the average caloric consumption (2,754 kcal) of the Spanish population [13].Although there are no significant differences were observed due to age, the caloric intakes are significantly lower in MTF groups of < 19 and > 50 years old, as a result of either psychological causes (group < 19 years old) or the assumption of their individual identity (group > 50 years old). However, if it is compared with the Spanish population, it is observed that women, relatively, intake more caloric diets than men [13].

The nutrient intake of this population makes them especially susceptible to chronic diseases. A significant proportion of the population studied therefore presents cardiovascular risk factors such as high intake of lipids, cholesterol and saturated fatty acids, as well as Na. This risk may be offset by the consumption of vitamins (C, E, A) and minerals (Se, Mn) that form part of the antioxidant system that protects against oxidative stress related to metabolic abnormalities associated with cardiovascular disease [14]. Likewise, the Farmer team has detected an increased risk of cardiovascular disease in sexual-minority women [15].

Although the number of meals depends on individual habits, lifestyles, working hours, etc., a good breakfast and lunch and a light dinner are recommended. This does not happen in the study population because only 13% have five meals a day and 16% skip breakfast. Finally, physical activity influences food consumption, and does so in different ways depending on gender. In those individuals who have become women, there is a direct relationship between energy consumption and energy consumed in their professional lives or by doing gymnastics or sport. The opposite occurs in those who have become men, who consume most energy while watching television, perhaps because they do so while consuming snacks or ice cream.

## 5. Conclusion

The gender dysphoria population studied generally suffers a change on his nutritional status with a larger overweight and obesity prevalence's. The diet is not healthy as a result of bad habits and lifestyle. It is therefore necessary to organize nutrition education courses in which they should be given the guidelines for a healthy lifestyle.
